# Anxiety and Depression Are Related to Higher Activity of Sphingolipid Metabolizing Enzymes in the Rat Brain

**DOI:** 10.3390/cells9051239

**Published:** 2020-05-17

**Authors:** Iulia Zoicas, Christiane Mühle, Anna K. Schmidtner, Erich Gulbins, Inga D. Neumann, Johannes Kornhuber

**Affiliations:** 1 Department of Psychiatry and Psychotherapy, Friedrich-Alexander University Erlangen-Nürnberg (FAU), 91054 Erlangen, Germany; Christiane.Muehle@uk-erlangen.de (C.M.); Johannes.Kornhuber@uk-erlangen.de (J.K.); 2 Department of Behavioural and Molecular Neurobiology, University of Regensburg, 93040 Regensburg, Germany; Anna-Kri.Schmidtner@mail.huji.ac.il (A.K.S.); inga.neumann@ur.de (I.D.N.); 3 Edmond and Lily Safra Center for Brain Sciences, Hebrew University of Jerusalem, Jerusalem 9190401, Israel; 4 Department of Molecular Biology, University of Duisburg-Essen, 45147 Essen, Germany; erich.gulbins@uni-due.de

**Keywords:** anxiety, depression, sphingolipids, sphingomyelinase, ceramidase

## Abstract

Changes in sphingolipid metabolism have been suggested to contribute to the pathophysiology of major depression. In this study, we investigated the activity of acid and neutral sphingomyelinases (ASM, NSM) and ceramidases (AC, NC), respectively, in twelve brain regions of female rats selectively bred for high (HAB) versus low (LAB) anxiety-like behavior. Concomitant with their highly anxious and depressive-like phenotype, HAB rats showed increased activity of ASM and NSM as well as of AC and NC in multiple brain regions associated with anxiety- and depressive-like behavior, including the lateral septum, hypothalamus, ventral hippocampus, ventral and dorsal mesencephalon. Strong correlations between anxiety-like behavior and ASM activity were found in female HAB rats in the amygdala, ventral hippocampus and dorsal mesencephalon, whereas NSM activity correlated with anxiety levels in the dorsal mesencephalon. These results provide novel information about the sphingolipid metabolism, especially about the sphingomyelinases and ceramidases, in major depression and comorbid anxiety.

## 1. Introduction

Major depressive disorder (MDD) is a severe and chronic mood disorder, with a lifetime prevalence of approximately 11% in men and 18% in women [1]. MDD is highly comorbid with other psychiatric disorders, including generalized anxiety, social anxiety and alcohol use disorders, and is associated with increased suicidality [2]. The major symptoms of MDD are a depressed mood and loss of interest, anhedonia, feelings of worthlessness, weight loss and insomnia. Despite its severity and high prevalence, the pathogenesis of MDD is yet unclear. One of the mechanisms involved in the pathogenesis of MDD seems to relate to an altered metabolism of sphingolipids such as sphingomyelin and ceramide [3]. The enzyme sphingomyelinase catalyzes the hydrolysis of sphingomyelin to ceramide and phosphorylcholine [4]. Depending on the pH optimum of the enzyme, several isoforms are known, including acid sphingomyelinase (ASM), neutral sphingomyelinase (NSM) and alkaline sphingomyelinase [5]. As expected from their pH optimum, these enzymes are differentially localized throughout the body. As such, ASM is ubiquitously distributed in all tissues [6] with a lysosomal form but is also constitutively secreted [7]. NSM is predominantly localized in the central nervous system [8,9], and alkaline sphingomyelinase is active in the digestive system but not in the central nervous system [10]. Similar to sphingomyelinases, ceramidases differ in their pH optimum for the breakdown of ceramide to sphingosine and fatty acid. The acid ceramidase (AC) and neutral ceramidase (NC) show much higher activities in the brain compared to peripheral tissues [9]. An alkaline isoform was detected in the human cerebellum [11].

Several clinical studies reported an altered sphingomyelin and ceramide metabolism in MDD. As such, ASM activity was increased in peripheral blood mononuclear cells of patients experiencing a major depressive episode [12]. Moreover, secretory ASM activity was related to depression severity and predicted the improvement of depressive symptoms during therapy [13]. Such alterations in ASM activity might involve changes in alternative splicing of the gene coding for ASM, which differed between MDD patients and healthy controls [14,15]. Similarly, several plasma ceramide species were increased in patients experiencing a major depressive episode during the past two years compared with healthy controls and patients experiencing a major depressive episode for more than two years [16]. Higher plasma ceramide Cer16:0, Cer18:0, Cer20:0, Cer22:0, Cer24:0 and Cer24:1 levels were also observed in patients with MDD and bipolar disorder [17], and higher plasma levels of ceramide Cer16:0 and Cer18:0 were associated with higher severity of depression symptoms in patients with coronary artery disease [18]. In contrast, plasma sphingomyelin SM26:1 [19], SM39:1 and SM39:2 [20] levels were decreased in MDD patients, and the SM23:1/SM16:0 ratio was negatively correlated with the severity of depression symptoms in a Dutch family-based lipidomics study [21].

Similarly increased ASM activity resulting in decreased sphingomyelin and increased ceramide concentrations was described in rodent models of MDD. For example, exposure to chronic unpredictable stress, which was shown to induce a depressive-like and anxious phenotype [22,23], increased the levels of several ceramide species in the hippocampus and frontal cortex but not in the amygdala and cerebellum in mice [24]. In contrast, sphingomyelin concentration was reduced by chronic unpredictable stress [24], suggesting an altered sphingolipid metabolism. Chronic administration of corticosterone, another model known to induce a depressive-like and anxious phenotype [25,26,27], also increased ceramide concentrations in the dorsal and ventral hippocampus [28]. The direct involvement of ceramide in the pathogenesis of depression was demonstrated in naïve mice, which developed a depressive-like but not an anxious phenotype after infusion of Cer16:0 ceramide into the dorsal hippocampus, whereas infusion of Cer8:0 or Cer20:0 ceramide exhibited no effect [29,30]. Interestingly, infusion of Cer16:0 ceramide into the basolateral amygdala induced an anxious but not a depressive-like phenotype in mice [30], demonstrating the complex species- and brain-region-specific modulation of emotional behavior by ceramide. Significant associations between ASM activity and depressive-like behavior were also described in transgenic mice overexpressing ASM throughout the body (ASMtg). These ASMtg mice showed an increased serum and hippocampal ASM activity and an increased hippocampal ceramide concentration that was associated with a depressive-like and anxious phenotype [29,31,32]. Interestingly, female but not male ASMtg mice also showed a social anxious phenotype [31], suggesting an association between increased ASM activity and deficits in social behavior in females. Sex-specific and brain-regional effects of ASM activity on emotional behavior were also demonstrated in conditional transgenic mice in which the overexpression of ASM was restricted to the forebrain (ASMtg^fb^). In these ASMtg^fb^ mice, males showed higher ASM activity in the frontal cortex, hippocampus, lateral septum and amygdala that resulted in a depressive-like phenotype, whereas females showed a higher ASM activity in the hypothalamus and a social anxious phenotype but not a depressive-like phenotype [33]. Despite this improved understanding of the effects of ASM and ceramide on MDD, little is known about the involvement of other sphingomyelinases and ceramidases in the pathophysiology of MDD or anxiety [34].

In this study, we characterized the brain activity of ASM and NSM as well as of AC and NC in an animal model of innate hyper-anxiety and MDD, namely, in Wistar rats selectively bred for extremely high (HAB) versus low (LAB) anxiety-like behavior, based on their performance on the elevated plus maze (EPM). This model is highly relevant for studying the sphingolipid metabolizing enzymes, as HAB rats show an anxious and depressive-like phenotype similar to ASMtg and ASMtg^fb^ mice but not a social anxious phenotype [35,36]. On the other hand, LAB rats show a low level of anxiety and depressive-like behavior when compared with HAB rats and unselected Wistar rats [37]. Given that MDD is more prevalent in women [38] and the social anxious phenotype was observed only in female ASMtg [31] and ASMtg^fb^ mice [33], experiments were performed in female HAB and LAB rats.

## 2. Materials and Methods

### 2.1. Animals

Female HAB and LAB rats were bred at the University of Regensburg. Rats were kept in colonies of 4 rats per cage under standard laboratory conditions (12:12 light-dark cycle, lights on at 06:00, 22 °C, 60% humidity, food and water ad libitum). Experiments were performed during the light phase in accordance to the Guide for the Care and Use of Laboratory Animals of the Government of Unterfranken (project identification code: 55.2-2532-2-384, approved on 06.04.2017) and the Guidelines of the NIH.

### 2.2. Experimental Design

At 9 weeks of age, the anxiety-like behavior of the rats was tested in the EPM. At 11 weeks of age, the naturally occurring social preference of the rats, as an indicator of social anxiety-like behavior, was tested in the social preference test (SPT). One week later, the depressive-like behavior of the rats was tested in the novelty-suppressed feeding paradigm (NSF). Twenty-four hours later, the rats were rapidly decapitated under CO_2_ anesthesia and their brains were removed, snap frozen and stored at −80 °C until further analysis. Twelve brain regions (i.e., the frontal cortex, dorsal and ventral striatum, lateral septum, amygdala, dorsal and ventral hippocampus, thalamus, hypothalamus, dorsal and ventral mesencephalon and cerebellum) were dissected out of coronal brain slices based on previous studies [30,39]. The activities of ASM and NSM as well as of AC and NC were analyzed from one hemisphere, counterbalanced between rats.

Experiments were performed in two batches of rats. In the first batch (*n* = 14 HAB rats and *n* = 15 LAB rats), all behavioral tests were performed as described above. In the second batch (*n* = 8 HAB rats and *n* = 8 LAB rats), the anxiety-like behavior was tested on the EPM at 9 weeks of age, and brains were collected at a comparable time point to the first batch. The activities of ASM, NSM, AC and NC were analyzed from both batches combined.

### 2.3. Elevated Plus-Maze Test (EPM)

The anxiety-like behavior of the rats was tested in the EPM as previously described [36]. The EPM consisted of two closed arms (50 × 10 × 40 cm; 10 lx) and two open arms (50 × 10 cm; 40 lx) connected through a central neutral zone (10 × 10 cm) elevated 70 cm from the floor. Rats were placed into the neutral zone facing one closed arm, and the 5-min test was recorded. A decreased percentage of time spent on the open arms indicated increased anxiety-like behavior.

### 2.4. Social Preference Test (SPT)

The naturally occurring social preference of the rats, as an indicator of social anxiety-like behavior, and the preference for social novelty of the rats, as an indicator of social recognition, were tested in the SPT as previously described [31,40]. Rats were placed in a novel arena (80 × 40 × 40 cm) and allowed to habituate for 30 s. Two identical wire-mesh cages (20 × 9 × 9 cm) were simultaneously placed at opposite side-walls of the arena for 5 min. One cage remained empty, and one cage contained an age-matched unfamiliar female rat (same rat). The initial position of the same rat varied between experimental rats to prevent possible place preference. After 5 min, the empty cage was exchanged by an identical cage containing a novel female rat for an additional 5 min. Experiments were recorded, and the time spent investigating (sniffing) the empty cage, the same and the novel rat was analyzed using JWatcher (Version 1.0, Macquarie University and UCLA). A higher investigation time directed toward the same rat versus the empty cage during the first 5 min indicated social preference and thus a lack of social anxiety. A higher investigation time directed toward the novel versus the same rat during the second 5 min indicated social recognition and preference for social novelty.

### 2.5. Novelty-Suppressed Feeding Paradigm (NSF)

The depressive-like behavior of rats was tested in the NSF as previously described [30,31]. Rats were food-deprived for 24 h prior to testing with unlimited water access. Rats were placed in a novel arena (80 × 80 × 40 cm) with the head facing one of the corners. Immediately afterwards, a single food pellet (ssniff Spezialdiäten GmbH, Soest, Germany) was placed in the center of the arena. The latency to feed, defined as biting the food pellet for longer than 3 s, was manually analyzed. An increased feeding latency indicated depressive-like behavior. The test lasted maximally 20 min. HAB rats that did not feed within these 20 min (56% of rats) were not removed from the study but were allocated 1200 s as their feeding latency value.

### 2.6. Measurement of Sphingomyelinase and Ceramidase Activities

The activity of sphingolipid metabolizing enzymes was determined using the fluorescent substrate BODIPY-FL-C12-SM (*N*-(4,4-difluoro-5,7-dimethyl-4-bora-3a,4a-diaza-s-indacene-3-dodecanoyl)sphingosylphosphocholine, D-7711, Thermo Fisher Scientific, Waltham, MA, USA) for sphingomyelinases and NBD-C12-ceramide (Cayman, obtained from Biomol, Hamburg, Germany) for ceramidases, with four replicates for each sample based on a previously established method [41]. Tissues were homogenized in lysis reagent (250 mM sucrose, 1 mM EDTA, 0.2% Triton X-100, 1× Roche protease inhibitor cocktail; approximately 200 μL/10 mg tissue) using a Tissue Lyser LT bead mill (Qiagen) with steal beads followed by freezing at −80 °C to enhance lysis. Supernatants obtained after thawing, ultrasound treatment (water bath for 60 s) and centrifugation at 16,000× *g* at 4 °C for 10 min were diluted 1:4 in lysis reagent and used for activity assays and for protein determination (Bradford/Coomassie kit, Thermo Fisher Scientific, Waltham, MA, USA). A standard enzyme reaction in a 96-well polystyrene plate contained 58 pmol sphingomyelin or 50 pmol ceramide as a substrate in a total volume of 50 μL of reaction buffer of the following composition: 200 mM sodium acetate buffer (pH 5.0), 500 mM NaCl, 0.2% IGEPAL^®^ CA-630 (NP 40) detergent for ASM, 200 mM HEPES buffer (pH 7.0), 200 mM MgCl_2_, 0.05% IGEPAL^®^ CA-630 (NP 40) for NSM; 200 mM sodium acetate buffer (pH 4.5), 100 mM NaCl, 0.03% IGEPAL^®^ CA-630 (NP 40) for AC and 200 mM HEPES (pH 7.0), 100 mM NaCl, 0.03% IGEPAL^®^ CA-630 (NP 40) for NC. The reaction was initiated by the addition of 2 μL of tissue lysate corresponding to 0.5–1 μg protein. After incubation at 37 °C for 1–18 h, depending on enzymatic activity, reactions were stopped by freezing at −20 °C and stored until further processing. To separate product and uncleaved substrate, 1.5 μL of each reaction were directly spotted on silica gel 60 thin layer chromatography plates (ALUGRAM SIL G, 818232, Macherey–Nagel, Düren, Germany) and separated using ethyl acetate with 1% (*v*/*v*) acetic acid as a solvent. Signals were detected on a Typhoon Trio scanner (488 nm excitation, 520 nm emission, 325–385 V, 100 μm resolution, GE Healthcare Life Sciences, Buckinghamshire, UK) and quantified with the ImageQuant software (GE Healthcare Life Sciences, Buckinghamshire, UK). Enzymatic activities were calculated as the hydrolysis rate of sphingomyelin or ceramide (pmol), respectively, per time (h) and per protein (μg).

### 2.7. Statistical Analysis

For statistical analysis, SPSS (Version 21, SPSS Inc., Chicago, IL, USA) was used. Data were analyzed using the Student t-test and two-way ANOVA for repeated measures, followed by a Bonferroni post-hoc analysis whenever appropriate. Spearman correlations were calculated to evaluate associations between behavior and enzyme activities within groups. Statistical significance was set at *p* < 0.05. For each parameter, outliers deviating more than two standard deviations from the mean were excluded from analysis. Graphs were prepared using GraphPad Prism 7.00 (GraphPad Software Inc., San Diego, CA, USA).

## 3. Results

### 3.1. Behavioral Phenotype of HAB and LAB Females

**Figure 1:**
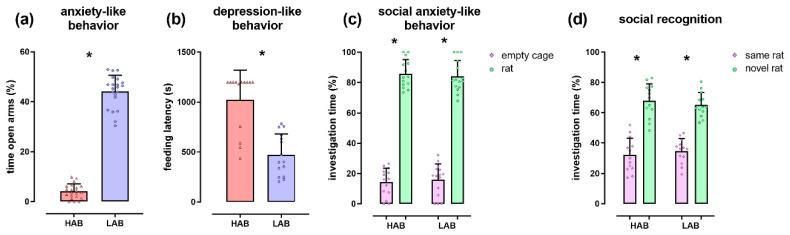
The behavioral phenotype of female HAB and LAB rats. (**a**) Percentage of time spent on the open arms of the elevated plus-maze, as an indicator of anxiety-like behavior; (**b**) Feeding latency shown in the novelty-suppressed feeding paradigm, as an indicator of depressive-like behavior; (**c**,**d**) Percentage of investigation time of the empty cage, the same and the novel female rat shown during the first (**c**) and second (**d**) 5 min of the social preference test, as indicators of social anxiety-like behavior and social recognition, respectively. Female HAB rats showed an anxious and depressive-like phenotype compared with LAB rats but unaltered social anxiety and social recognition. Data represent individual data points with means ± SEM. * *p* < 0.05.

The expected highly anxious and depressive-like phenotype of HAB females compared with LAB females was visible in both the EPM (T(40) = −40.02; *p* < 0.001; Figure 1a) and NSF (T(26) = 5.63; *p* < 0.001; Figure 1b), respectively. In the SPT, all rats displayed normal social preference and a lack of social anxiety (stimulus effect F(1,52) = 246.64; *p* < 0.001; group × stimulus effect F(1,52) = 1.90; *p* = 0.174; Figure 1c). Furthermore, all rats displayed normal social recognition and preference for social novelty (stimulus effect F(1,52) = 44.38; *p* < 0.001; group × stimulus effect F(1,52) = 0.43; *p* = 0.514; Figure 1d).

### 3.2. Enzyme Activities in Selected Brain Regions of HAB and LAB Females

**Figure 2:**
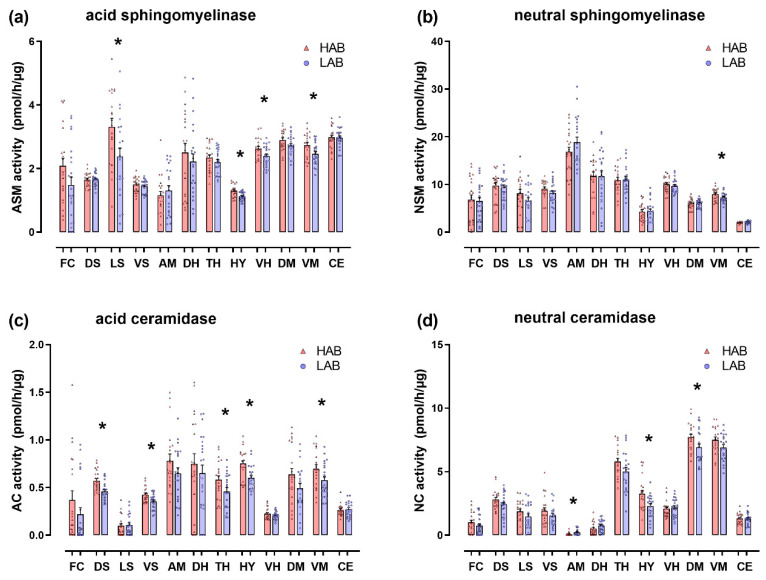
The activity of sphingolipid metabolizing enzymes in brains of female HAB and LAB rats. The activity of acid and neutral sphingomyelinases (ASM in (**a**), NSM in (**b**)) as well as of acid and neutral ceramidases (AC in (**c**), NC in (**d**)) was analyzed in the frontal cortex (FC), dorsal striatum (DS), lateral septum (LS), ventral striatum (VS), amygdala (AM), dorsal hippocampus (DH), thalamus (TH), hypothalamus (HY), ventral hippocampus (VH), dorsal mesencephalon (DM), ventral mesencephalon (VM) and cerebellum (CE). HAB rats showed a significant increase in the activity of these enzymes compared with LAB rats. Data represent individual data points with means ± SEM. * *p* < 0.05.

HAB females showed an increased ASM activity in the lateral septum (+40%, T(40) = 2.52; *p* = 0.016), hypothalamus (+15%, T(42) = 3.40; *p* = 0.001), ventral hippocampus (+10%, T(41) = 2.31; *p* = 0.026) and ventral mesencephalon (+11%, T(41) = 2.42; *p* = 0.020) compared with LAB females, whereas no differences in ASM activity were found in the frontal cortex, amygdala, dorsal hippocampus, dorsal and ventral striatum, dorsal mesencephalon, thalamus and cerebellum (Figure 2a). HAB females showed an increased NSM activity in the ventral mesencephalon (+10%, T(39) = 2.08; *p* = 0.044) but no significant differences in the other brain regions (Figure 2b). HAB females also showed an increased AC activity in the dorsal (+24%, T(43) = 3.69; *p* < 0.001) and ventral (+20%, T(42) = 2.98; *p* = 0.005) striatum, hypothalamus (+25%, T(41) = 3.50; *p* = 0.001), thalamus (+27%, T(43) = 2.18; *p* = 0.035) and ventral mesencephalon (+21%, T(41) = 2.10; *p* = 0.042) (Figure 2c) and an increased NC activity in the hypothalamus (+42%, T(41) = 2.84; *p* = 0.007) and dorsal mesencephalon (+13%, T(41) = 2.37; *p* = 0.022) compared with LAB females (Figure 2d). The only enzymatic activity that was decreased in HAB females compared with LAB females was the NC activity in the amygdala (−62%, T(42) = −2.47; *p* = 0.019) (Figure 2d).

### 3.3. Correlations between Behavior and Enzyme Activities in HAB and LAB Females

In addition to the observed group differences, enzyme activities were also related to depression- and anxiety-like behavior within the groups. The anxiety-like behavior expressed as percentage time spent on the open arms of the EPM negatively correlated with ASM activity in the amygdala (*r* = −0.48; *p* = 0.031), ventral hippocampus (*r* = −0.57; *p* = 0.008) and dorsal mesencephalon (*r* = −0.60; *p* = 0.006) and with NSM activity in the dorsal mesencephalon (*r* = −0.64; *p* = 0.002) in HAB females but with none of the parameters in LAB females.

Depressive-like behavior expressed as latency to feed after a 24-h food deprivation period in the NSF negatively correlated with AC activity in the frontal cortex in LAB females (*r* = −0.62; *p* = 0.015) but with none of the parameters in HAB females. This lower number of correlations between enzyme activities and depressive- versus anxiety-like behavior might be due to the lower number of rats in which depressive-like behavior was assessed (*n* = 29 versus *n* = 45).

## 4. Discussion

Our study describes for the first time the activity of sphingomyelinases and ceramidases in the brain of rats with high and low levels of innate anxiety- and depressive-like behavior. Concomitant with their highly anxious and depressive-like phenotype, HAB rats showed increased activity of ASM and NSM as well as of AC and NC in multiple brain regions associated with anxiety- and depressive-like behavior, including the lateral septum, hypothalamus, ventral hippocampus and ventral and dorsal mesencephalon. 

These findings extend previous reports of increased brain ASM activity in transgenic models of MDD, namely, in ASMtg [29] and ASMtg^fb^ mice [33]. As such, increased ASM activity was previously described in the dorsal and ventral hippocampus in ASMtg mice overexpressing ASM in the whole body [29] (no other brain regions were tested in this study), whereas in ASMtg^fb^ mice overexpressing ASM only in the forebrain [33], ASM activity was increased in multiple brain regions. As such, both male and female ASMtg^fb^ mice showed increased ASM activity in the dorsal striatum, dorsal and ventral hippocampus and amygdala compared with wild-type controls. Furthermore, male ASMtg^fb^ mice showed higher ASM activity compared with female ASMtg^fb^ mice in all these regions except in the dorsal striatum where the ASM activity was similarly high in both male and female ASMtg^fb^ mice. In the hypothalamus, the activity of ASM was increased in female but not in male ASMtg^fb^ mice, whereas in the frontal cortex, lateral septum and ventral mesencephalon, the ASM activity was increased in male but not in female ASMtg^fb^ mice. Despite this increased ASM activity, female ASMtg^fb^ mice did not show an anxious and depressive-like phenotype [33]. In contrast, the behavior of the male ASMtg^fb^ mice and female HAB rats clearly changed, displaying a significant anxious and depressive-like phenotype in the EPM and NSF, respectively. By directly comparing these two animal models of MDD with similar behavioral deficits, an important role of the lateral septum and ventral mesencephalon in anxiety- and depressive-like behavior might be suggested, as female HAB rats and male ASMtg^fb^ mice but not female ASMtg^fb^ mice showed an increased ASM activity within these brain areas. Given that the lateral septum plays a critical role in regulating processes related to mood and motivation [42,43] and is neuroanatomically connected with several brain regions known to regulate emotional behavior (e.g., the hippocampus, amygdala, hypothalamus and the mesolimbic system [44]), it is feasible that ASM within the lateral septum and ventral mesencephalon regulates anxiety- and depressive-like behavior. The ASM within the amygdala, ventral hippocampus and dorsal mesencephalon might also regulate anxiety- and depressive-like behavior given that a negative correlation between anxiety levels and ASM activity was found in these brain regions in female HAB rats, i.e., rats that spent less time on the open arms of the EPM and were thereby more anxious also showed higher levels of ASM activity in the amygdala, ventral hippocampus and dorsal mesencephalon. However, the increased ASM activity within the amygdala and ventral hippocampus in female ASMtg^fb^ mice did not result in an anxious and depressive-like phenotype [33], suggesting a differential contribution of the amygdalar and ventral hippocampal ASM to anxiety- and depressive-like behavior in these two animal models of MDD. An indirect involvement of amygdalar ASM in anxiety-like behavior has been already suggested in a previous study that showed that infusion of Cer16:0 ceramide into the basolateral amygdala induced an anxious phenotype in mice [30]. As ceramide is generated by the activity of ASM, a higher ASM activity might be expected to increase ceramide levels. It is also possible that a very high level of ASM activity within the amygdala and ventral hippocampus, as seen in male but not in female ASMtg^fb^ mice [33], might be necessary to elicit changes in anxiety- and depressive-like behavior in this transgenic mouse model.

We also aimed to identify the brain region/s contributing to ASM effects on social anxiety by comparing the alterations in ASM activity between female ASMtg^fb^ mice with social anxiety and female HAB rats without social anxiety. However, for now we cannot suggest which brain region mediates these effects. Based on the study performed in ASMtg^fb^ mice, the hypothalamus was a promising brain region given that the ASM activity was increased in female but not in male ASMtg^fb^ mice and that only female ASMtg^fb^ mice showed a social anxious phenotype [33]. Although the hypothalamus is highly relevant for several types of social behavior, including social anxiety [45], it is unlikely that the increased ASM activity within the hypothalamus contributes to social anxiety in general, as female HAB rats were not socially anxious despite the increased ASM activity in the hypothalamus. In female HAB rats, the increased ASM activity in the hypothalamus seems to relate more to their depressive-like behavior, given that a tendency towards a positive correlation between depressive-like behavior and ASM activity was found (*r* = 0.54; *p* = 0.056). As such, rats that showed higher feeding latencies in the NSF and thereby a more severe depressive-like behavior tended to show higher levels of ASM activity in the hypothalamus. A more detailed analysis of ASM activity within distinct hypothalamic nuclei might reveal whether specific nuclei mediate the effects of ASM on social anxiety. Alternatively, a high level of ASM activity in other brain regions such as the thalamus and/or ventral striatum might be necessary to induce social anxiety, given that several studies suggested an important role of the thalamus [46,47] and the ventral striatum [48,49] in the pathophysiology of social anxiety and that ASM activity was unaltered in these brain regions in female HAB rats. 

Although previous studies investigated the activity of ASM in depressive patients [12] and in animal models of MDD [29,33], little is known about the contribution of other enzymes of the sphingolipid metabolism such as NSM, AC and NC in MDD. We showed that the activity of NSM was also increased in the ventral mesencephalon in female HAB rats, similar to ASM, suggesting that ASM and NSM might regulate anxiety- and depressive-like behavior together within this brain region. Similar to ASM, NSM within the dorsal mesencephalon might also regulate anxiety-like behavior in female HAB rats given that a negative correlation between anxiety levels and NSM activity was observed, i.e., rats that spent less time on the open arms of the EPM and were thereby more anxious also showed higher levels of NSM activity in the dorsal mesencephalon. 

As ceramide is generated by the activity of ASM and NSM, an increase in ASM and NSM activity levels is expected to increase ceramide levels and therefore also increase the activity of the enzymes metabolizing ceramide, i.e., AC and NC. In agreement with this hypothesis, we found an increased AC activity in the dorsal and ventral striatum, thalamus, hypothalamus and ventral mesencephalon, whereas the activity of NC was increased in the hypothalamus and dorsal mesencephalon. However, whether this relatively small but significantly increased activity of ASM and NSM is physiologically relevant and leads to increased ceramide levels remains to be verified. Mass spectrometry of brain-specific tissue samples could reveal such alterations in sphingolipid and ceramide species of different chain length. Moreover, new label-free imaging mass spectrometry techniques could visualize the distribution and provide quantitative data on various subtypes of lipids in these tissue sections [32]. In addition, future studies could include mass spectrometry as well as enzyme activity assays of cerebrospinal fluid samples with detectable levels of both ASM [50] and NSM [51]. Further enzymes involved in regulating ceramide levels and known to be affected in neuropsychiatric disorders (e.g., alkaline ceramidase and sphingomyelin synthase [52]) might also play a role and could be altered in specific brain regions, counterbalancing or increasing the effects of the analyzed sphingomyelinases and ceramidases. 

Taken together, our results add a novel piece of information to the complex regulation of sphingolipid metabolism, especially of the sphingomyelinases and ceramidases, in MDD and demonstrate that all investigated enzymes (i.e., ASM, NSM, AC and NC) show predominantly an increased activity in multiple brain regions associated with anxiety- and depressive-like behavior.

## References

[1] World Health Organization (2017). Depression and Other Common Mental Disorders: Global Health Estimates.

[2] Lenz B., Rother M., Bouna-Pyrrou P., Mühle C., Tektas O.Y., Kornhuber J. (2019). The androgen model of suicide completion. Prog. Neurobiol..

[3] Müller C.P., Reichel M., Mühle C., Rhein C., Gulbins E., Kornhuber J. (2015). Brain membrane lipids in major depression and anxiety disorders. Biochim. Biophys. Acta.

[4] Schneider P.B., Kennedy E.P. (1967). Sphingomyelinase in normal human spleens and in spleens from subjects with Niemann-Pick disease. J. Lipid Res..

[5] Goni F.M., Alonso A. (2002). Sphingomyelinases: Enzymology and membrane activity. FEBS Lett..

[6] Weinreb N.J., Brady R.O., Tappel A.L. (1968). The lysosomal localization of sphingolipid hydrolases. Biochim. Biophys. Acta.

[7] Kornhuber J., Rhein C., Müller C.P., Mühle C. (2015). Secretory sphingomyelinase in health and disease. Biol. Chem..

[8] Rao B.G., Spence M.W. (1976). Sphingomyelinase activity at pH 7.4 in human brain and a comparison to activity at pH 5.0. J. Lipid Res..

[9] Mühle C., Reichel M., Gulbins E., Kornhuber J. (2013). Sphingolipids in psychiatric disorders and pain syndromes. Handb. Exp. Pharm..

[10] Duan R.D., Hertervig E., Nyberg L., Hauge T., Sternby B., Lillienau J., Farooqi A., Nilsson A. (1996). Distribution of alkaline sphingomyelinase activity in human beings and animals. Tissue and species differences. Dig. Dis. Sci..

[11] Sugita M., Willians M., Dulaney J.T., Moser H.W. (1975). Ceramidase and ceramide synthesis in human kidney and cerebellum. Description of a new alkaline ceramidase. Biochim. Biophys. Acta.

[12] Kornhuber J., Medlin A., Bleich S., Jendrossek V., Henkel A.W., Wiltfang J., Gulbins E. (2005). High activity of acid sphingomyelinase in major depression. J. Neural Transm. (Vienna).

[13] Mühle C., Wagner C.J., Färber K., Richter-Schmidinger T., Gulbins E., Lenz B., Kornhuber J. (2019). Secretory acid sphingomyelinase in the serum of medicated patients predicts the prospective course of depression. J. Clin. Med..

[14] Rhein C., Reichel M., Kramer M., Rotter A., Lenz B., Mühle C., Gulbins E., Kornhuber J. (2017). Alternative splicing of *SMPD1* coding for acid sphingomyelinase in major depression. J. Affect. Disord..

[15] Rhein C., Tripal P., Seebahn A., Konrad A., Kramer M., Nagel C., Kemper J., Bode J., Mühle C., Gulbins E. (2012). Functional implications of novel human acid sphingomyelinase splice variants. PLoS ONE.

[16] Gracia-Garcia P., Rao V., Haughey N.J., Bandaru V.V., Smith G., Rosenberg P.B., Lobo A., Lyketsos C.G., Mielke M.M. (2011). Elevated plasma ceramides in depression. J. Neuropsychiatry Clin. Neurosci..

[17] Brunkhorst-Kanaan N., Klatt-Schreiner K., Hackel J., Schroter K., Trautmann S., Hahnefeld L., Wicker S., Reif A., Thomas D., Geisslinger G. (2019). Targeted lipidomics reveal derangement of ceramides in major depression and bipolar disorder. Metabolism.

[18] Dinoff A., Saleem M., Herrmann N., Mielke M.M., Oh P.I., Venkata S.L.V., Haughey N.J., Lanctot K.L. (2017). Plasma sphingolipids and depressive symptoms in coronary artery disease. Brain Behav..

[19] Moaddel R., Shardell M., Khadeer M., Lovett J., Kadriu B., Ravichandran S., Morris P.J., Yuan P., Thomas C.J., Gould T.D. (2018). Plasma metabolomic profiling of a ketamine and placebo crossover trial of major depressive disorder and healthy control subjects. Psychopharmacol (Berlin).

[20] Liu X., Li J., Zheng P., Zhao X., Zhou C., Hu C., Hou X., Wang H., Xie P., Xu G. (2016). Plasma lipidomics reveals potential lipid markers of major depressive disorder. Anal. Bioanal. Chem..

[21] Demirkan A., Isaacs A., Ugocsai P., Liebisch G., Struchalin M., Rudan I., Wilson J.F., Pramstaller P.P., Gyllensten U., Campbell H. (2013). Plasma phosphatidylcholine and sphingomyelin concentrations are associated with depression and anxiety symptoms in a Dutch family-based lipidomics study. J. Psychiatr. Res..

[22] Willner P. (2017). The chronic mild stress (CMS) model of depression: History, evaluation and usage. Neurobiol. Stress.

[23] Zhou X.D., Shi D.D., Zhang Z.J. (2019). Antidepressant and anxiolytic effects of the proprietary Chinese medicine Shexiang Baoxin pill in mice with chronic unpredictable mild stress. J. Food Drug Anal..

[24] Oliveira T.G., Chan R.B., Bravo F.V., Miranda A., Silva R.R., Zhou B., Marques F., Pinto V., Cerqueira J.J., Di Paolo G. (2016). The impact of chronic stress on the rat brain lipidome. Mol. Psychiatry.

[25] Gregus A., Wintink A.J., Davis A.C., Kalynchuk L.E. (2005). Effect of repeated corticosterone injections and restraint stress on anxiety and depression-like behavior in male rats. Behav. Brain Res..

[26] Murray F., Smith D.W., Hutson P.H. (2008). Chronic low dose corticosterone exposure decreased hippocampal cell proliferation, volume and induced anxiety and depression like behaviours in mice. Eur. J. Pharm..

[27] David D.J., Samuels B.A., Rainer Q., Wang J.W., Marsteller D., Mendez I., Drew M., Craig D.A., Guiard B.P., Guilloux J.P. (2009). Neurogenesis-dependent and -independent effects of fluoxetine in an animal model of anxiety/depression. Neuron.

[28] Miranda A.M., Bravo F.V., Chan R.B., Sousa N., Di Paolo G., Oliveira T.G. (2019). Differential lipid composition and regulation along the hippocampal longitudinal axis. Transl. Psychiatry.

[29] Gulbins E., Palmada M., Reichel M., Luth A., Bohmer C., Amato D., Muller C.P., Tischbirek C.H., Groemer T.W., Tabatabai G. (2013). Acid sphingomyelinase-ceramide system mediates effects of antidepressant drugs. Nat. Med..

[30] Zoicas I., Huber S.E., Kalinichenko L.S., Gulbins E., Müller C.P., Kornhuber J. (2019). Ceramides affect alcohol consumption and depressive-like and anxiety-like behavior in a brain region- and ceramide species-specific way in male mice. Addict. Biol..

[31] Zoicas I., Reichel M., Gulbins E., Kornhuber J. (2016). Role of acid sphingomyelinase in the regulation of social behavior and memory. PLoS ONE.

[32] Müller C.P., Kalinichenko L.S., Tiesel J., Witt M., Stockl T., Sprenger E., Fuchser J., Beckmann J., Praetner M., Huber S.E. (2017). Paradoxical antidepressant effects of alcohol are related to acid sphingomyelinase and its control of sphingolipid homeostasis. Acta Neuropathol..

[33] Zoicas I., Schumacher F., Kleuser B., Reichel M., Gulbins E., Fejtova A., Kornhuber J., Rhein C. (2020). The forebrain—Specific overexpression of acid sphingomyelinase induces depressive-like symptoms in mice. Cells.

[34] Kalinichenko L.S., Mühle C., Eulenburg V., Praetner M., Reichel M., Gulbins E., Kornhuber J., Müller C.P. (2019). Enhanced alcohol preference and anxiolytic alcohol effects in Niemann-Pick Disease model in mice. Front. Neurol..

[35] Liebsch G., Montkowski A., Holsboer F., Landgraf R. (1998). Behavioural profiles of two Wistar rat lines selectively bred for high or low anxiety-related behaviour. Behav. Brain Res..

[36] Schmidtner A.K., Slattery D.A., Glasner J., Hiergeist A., Gryksa K., Malik V.A., Hellmann-Regen J., Heuser I., Baghai T.C., Gessner A. (2019). Minocycline alters behavior, microglia and the gut microbiome in a trait-anxiety-dependent manner. Transl. Psychiatry.

[37] Wegener G., Mathe A.A., Neumann I.D. (2012). Selectively bred rodents as models of depression and anxiety. Curr. Top. Behav. Neurosci..

[38] Brivio E., Lopez J.P., Chen A. (2020). Sex differences: Transcriptional signatures of stress exposure in male and female brains. Genes Brain Behav..

[39] Huber S.E., Zoicas I., Reichel M., Mühle C., Buttner C., Ekici A.B., Eulenburg V., Lenz B., Kornhuber J., Müller C.P. (2018). Prenatal androgen receptor activation determines adult alcohol and water drinking in a sex-specific way. Addict. Biol..

[40] Lukas M., Toth I., Reber S.O., Slattery D.A., Veenema A.H., Neumann I.D. (2011). The neuropeptide oxytocin facilitates pro-social behavior and prevents social avoidance in rats and mice. Neuropsychopharmacology.

[41] Mühle C., Kornhuber J. (2017). Assay to measure sphingomyelinase and ceramidase activities efficiently and safely. J. Chromatogr. A.

[42] Sheehan T.P., Neve R.L., Duman R.S., Russell D.S. (2003). Antidepressant effect of the calcium-activated tyrosine kinase Pyk2 in the lateral septum. Biol. Psychiatry.

[43] Muigg P., Hoelzl U., Palfrader K., Neumann I., Wigger A., Landgraf R., Singewald N. (2007). Altered brain activation pattern associated with drug-induced attenuation of enhanced depression-like behavior in rats bred for high anxiety. Biol. Psychiatry.

[44] Sheehan T.P., Chambers R.A., Russell D.S. (2004). Regulation of affect by the lateral septum: Implications for neuropsychiatry. Brain Res. Brain Res. Rev..

[45] Choleris E., Devidze N., Kavaliers M., Pfaff D.W. (2008). Steroidal/neuropeptide interactions in hypothalamus and amygdala related to social anxiety. Prog. Brain Res..

[46] Vertes R.P., Linley S.B., Hoover W.B. (2015). Limbic circuitry of the midline thalamus. Neurosci. Biobehav. Rev..

[47] Wang X., Cheng B., Luo Q., Qiu L., Wang S. (2018). Gray matter structural alterations in social anxiety disorder: A voxel-based meta-analysis. Front. Psychiatry.

[48] Becker M.P.I., Simon D., Miltner W.H.R., Straube T. (2017). Altered activation of the ventral striatum under performance-related observation in social anxiety disorder. Psychol. Med..

[49] Gonzalez M.Z., Puglia M.H., Morris J.P., Connelly J.J. (2019). Oxytocin receptor genotype and low economic privilege reverses ventral striatum-social anxiety association. Soc. Neurosci..

[50] Mühle C., Huttner H.B., Walter S., Reichel M., Canneva F., Lewczuk P., Gulbins E., Kornhuber J. (2013). Characterization of acid sphingomyelinase activity in human cerebrospinal fluid. PLoS ONE.

[51] Sarrafpour S., Ormseth C., Chiang A., Arakaki X., Harrington M., Fonteh A. (2019). Lipid metabolism in late-onset Alzheimer’s disease differs from patients presenting with other dementia phenotypes. Int. J. Environ. Res. Public Health.

[52] Mühle C., Bilbao Canalejas R.D., Kornhuber J. (2019). Sphingomyelin synthases in neuropsychiatric health and disease. Neurosignals.

